# Effects of Appearance- and Performance-Enhancing Drugs on Personality Traits

**DOI:** 10.3389/fpsyt.2021.730167

**Published:** 2021-09-24

**Authors:** Simona Zaami, Adele Minutillo, Ascanio Sirignano, Enrico Marinelli

**Affiliations:** ^1^Department of Anatomical, Histological, Forensic and Orthopedic Sciences, Sapienza University, Rome, Italy; ^2^National Centre on Addiction and Doping, Istituto Superiore di Sanità, Rome, Italy; ^3^School of Law, Medico-Legal Section, University of Camerino, Camerino, Italy

**Keywords:** appearance- and performance-enhancing drugs, APEDs, anabolic–androgenic steroids, personality traits, psychiatric implication

## Abstract

Appearance- and performance-enhancing drugs (APEDs) are commonly used by adolescents and young adults in an effort to improve not only athletic performance but also physical and mental efficiency and sexual appearance. The rationale for using these drugs is grounded in the perceived importance of external appearance, the quest for health and youth, and the urge to boost one's sexual performances. Although APED users tend to be quite moderate overall, some specific subpopulations can display pathological use associated with high-risk behaviors. A wide and diverse range of APEDs is now easily accessible to almost anyone through backdoor online avenues. Common APEDs include anabolic–androgenic steroids, non-steroidal anabolics, anorectics, diuretics and ergo/thermogenics, nootropics or “cognition enhancers,” licit and illicit psychostimulants, and finally, sexual enhancers. The use of APEDs appears linked to several psychopathological disorders of unclear prevalence, e.g., body image disorders and eating disorders, perfectionism, but also depression and loneliness. The role of personality traits related to APED use has been investigated in adolescents and young adults, in elite and amateur athletes, and in chemsexers and associated with the above-reported personality traits. The studies herein analyzed show that APED consumption in the general population is quickly growing into a public health concern. It is therefore essential to launch prevention and intervention projects aimed at promoting safe instrumental use of the body, not only in sports disciplines but also among the general population, and to promote psychological aid procedures for people with substance use issues, depression and anxiety, and body image disorders.

## Introduction

The definition “appearance- and performance-enhancing drugs (APEDs)” started to be applied to drugs and supplements used to improve not only athletic appearance and performance but also cognitive and sexual performance.

The most widely misused APEDs include the following:

- Anabolic–androgenic steroids promoting bone strength and feelings of well-being, with consequent testosterone-supplementation therapy being used for mood and sexual performance problems associated with male aging (1–3);
- Non-steroidal anabolics (e.g., insulin, insulin-like growth factor, and human growth hormone, beta-2 adrenergic drugs) (1);
- Anorectics, diuretics, and ergo/thermogenics to lose weight and to decrease body fat or to promote thinness and increase muscle mass (1);
- Nootropics, or “cognition enhancers,” normally used to treat cognition deficits in individuals suffering from attention deficit hyperactivity disorder, senile dementia, Alzheimer's disease, etc., but misused to improve memory (e.g., increasing working memory capacity or updating) or other aspects of cognitive control (e.g., inhibitory control, attentional control, and attention span), such as methylphenidate, ethylphenidate, modafinil, dexamfetamine, rivastigmine, donezepil, memantine, and nootropic supplements (4, 5), and also some new psychoactive substances, such as phenibut used for this specific purpose (6–8); and
- “Chemsex” drugs, namely substances consumed immediately before or during sexual intercourses to facilitate, prolong, and/or intensify sexual experience such as methamphetamine, cocaine, mephedrone, gamma hydroxybutyric acid, gammabutyrolactone, ketamine, and poppers together with sildenafil and congeners (9–11).

The negative effects of APEDs on the physical health of users' have received considerable attention (12, 13). It has also been shown that most of the risk relative to APEDs is linked to long-term use (12) and also to the poly-misuse of the above-reported classes of APED drugs (anabolic–androgenic steroids with chemsex drugs, chemsex drugs and nootropics, etc.), with synergistic toxic effects on physical and psychic homeostasis.

In addition to physical side effects, the use of APEDs has been associated with psychological repercussions and unwanted side effects. The reported positive ones include, among others (13), perceived power over others, higher self-esteem, and better concentration. On the other side, APED consumption has been related to negative psychological consequences, such as depression, anxiety all the way to psychosis (14), and aggression (15–18). The harmful potential of some APEDs, anabolic–androgenic steroids in particular, stems from their capability to bring about long-term alterations in the neurotransmitter pathways of the brain of the user, in addition to functioning as androgen receptors; that means that such substances can affect both cellular functioning and gene expression. Specifically, steroids affect the brain serotonin and dopamine neurotransmitter systems, thus affecting the regulation of sleep patterns, appetite, sexuality, learning skills, movement, and emotions.

Dopamine is a key neurotransmitter for reward system regulation, which makes it pivotal in terms of developing addiction. Steroids such as nandrolone have been found to alter the dopamine system response to stimulating intoxicants, causing the release of neurotransmitters induced by such substances to decrease and the sense of reward obtained from them to dwindle as well (19). In some studies, androgenic compounds have been shown to have direct activating functions for dopamine and serotonin release. It is worth noting in that regard that activation of androgen receptors can give rise to abrupt increases in calcium levels in brain cells, skeletal muscle, and heart (20). Calcium itself is also heavily involved in neuronal signaling and in the transmission of depolarizing signals, thus contributing to synaptic activity (12, 21). Moreover, prolonged effects on the reproductive system of athletes and recreational users of steroids have been thoroughly documented: most AAS users have shown long-lasting hypogonadism (22) and low gonadotropin and testosterone levels (23, 24).

At any rate, the most recurrent personality traits correlated with APED use entail body image disturbance (13, 25) and personality changes (26), as well as symptoms stemming from abuse and dependence (27, 28).

This mini-review aims to briefly illustrate the role of perfectionism and body image in APED users and their personality traits, with a close focus on the most frequent APED consumers: youngsters and young adult users of cognitive enhancers, elite as well as amateur athletes, and chemsexers. A literature search was performed on the multidisciplinary research databases PubMed, Scopus, and Web of Science, to identify all the relevant updated articles. The search terms used in different combinations were as follows: appearance- and performance-enhancing drugs, APEDs, anabolic–androgenic steroids, personality traits, and psychiatric implication.

## Perfectionism and Body Image in APED Users

Perfectionism is a personality trait described by constant research of exceedingly high standards of personal physical and cognitive performance (29).

There are few specific studies about perfectionism and APED users, and as a function of the applied model to define perfectionism, contradictory results have been obtained in students using psychostimulants or in different classes of elite and recreational athletes (30, 31). In any case, as reported below, perfectionism as a personality trait is present in athletes using doping agents, students and young adults misusing cognitive enhancers, and chemsexers using stimulant drugs (32–34).

Body image is defined as the visual appearance we project outwardly, which calls for attention in social interactions (35), and is therefore deemed highly important for social approval and acceptance (36).

Engagement in sports or physical activities is an obvious way to achieve that goal, which can be used to show physical attractiveness and sex appeal alike. In this concern, APEDs are viewed as potential accelerators in developing a fit and attractive body and then maintaining the effects. Thus, APEDs can be considered as a means to bridge the gap between the desire to improve perceived body image and the difficulties in achieving such a goal.

APED consumption is better explained by the sense-making related to body image, rather than the cognitive evaluation of social norms about appearance and consequent psychopathology-oriented approach (37). Furthermore, an investigation focused on adolescents has found that the use of substances like nutritional supplements or anabolic steroids is linked to body dissatisfaction, feelings of sadness or hopelessness, and perceptions of overweight (38).

Indeed, the feeling of body dissatisfaction, starting from young age, remains relatively stable across the life span (39) and eventually decreases with age, even if it is well-known that another important issue linked to body image and APED use resides in the varying individual degrees of aging acceptance (40).

## Personality Traits in Cognitive Enhancer Users

Cognitive enhancers, also known as nootropics or “smart drugs” (41), comprise both dietary supplements and pharmacologically active substances mainly (but not only) misused by adolescents and young adults (42) for the purpose of achieving higher levels of alertness, attention and memory potential (43, 44), learning performance, creativity, and motivation (45).

Even though the so-called “nootropics” include a variety of over-the-counter preparations, freely sold on the internet (e.g., the racetams such as piracetam, phenylpiracetam, and analogs; cholinergics such as citicoline and choline bitartrate; herbal products such as *Bacopa monnieri, Panax ginseng*, and *Ginkgo biloba*), the most popular ones are central nervous system neurotransmitter stimulants (e.g., methylphenidate, modafinil, and amphetamine salt mixtures), normally prescribed for conditions such as attention deficit hyperactivity disorder, narcolepsy, Alzheimer's disease, dementia, and similar pathological conditions (46, 47).

The prevalence in the use of cognitive enhancers, typically determined in university students, varied from 4.2% in Brazilian students from different academic disciplines (48), to 6.2, 5.9, and 2% for modafinil, methylphenidate, and amphetamine use, respectively, among UK and Irish university students (49) to 8.1 and 8.7% Lithuanian and Iranian medical students, respectively, consuming modafinil, methylphenidate, and amphetamine (48, 50). Maximum values of prevalence use were found in US students, with an estimate range between 5 and 43% (51) and a mean value of 17% obtained in a meta-analysis study (52).

Among the personality traits related to the use of cognitive enhancers, it has been shown that consumers are more inclined toward the socially unacceptable methods of improving their achievement by manipulation or dishonesty such as APED intake (53).

Cognitive enhancement drug use bears a relationship with the dark triad personality traits: Machiavellianism, narcissism, and psychopathy (54). One reason for this association is rooted in the fact that people with high scores on the dark triad personality traits, especially psychopathy and narcissism, were demonstrated to be more prone to risk-taking, in the form of substance abuse including cognitive enhancers (55).

In addition, it is likely that people who use cognitive enhancement drugs tend to always strive for better personal social or working achievement, even in unfair ways (53). The endeavor for egoism, superiority, and manipulation is a hallmark feature of those scoring high on dark triad personality traits and consuming cognitive enhancers.

In other words, people with high scores on the dark triad might tend to have devious behavior for the achievement of personal benefits while disregarding potential adverse consequences for others. In this concern, APEDs and specifically cognitive enhancers use, which is also to some extent illegal and therefore unethical, can be seen as a form of cheating behavior in the furtherance of personal objectives, with total disregard for the possible health risks that might ensue. This gives rise to the assumption that the dark triad personality traits may be ascribed to cognitive enhancers use.

A study on the personal characteristics of cognitive enhancer consumers established that manipulation and insensitivity are factors in common with the three dark triad personality traits and this evidence explains the positive relation between the three dark triad personality traits and cognitive enhancers use (47).

## Personality Traits in Elite and Recreational Athletes Using APEDs

The use of pharmacologically active substances to enhance sport performance is commonly referred to as doping (56, 57).

In a context in which achieving victory and setting new records is increasingly difficult, several athletes choose to take shortcuts, cheating in order to prevail and/or set new records. Steroids are the drugs that often come to mind whenever doping is discussed, but doping also includes the use of other forbidden drugs (such as stimulants, hormones, diuretics, narcotics, and marijuana), use of forbidden methods (such as blood transfusions or gene doping), and even the refusal to take a drug test or any attempt to tamper with doping controls.

Indeed, since man has always tried to improve his performance, the doping issue has apparently existed ever since sports activities became a large-scale social phenomenon.

Although until recently this practice only involved elite athletes, scientific evidence shows that the use of doping outside elite sports has been steadily rising as an emerging public health challenge (58).

The role of personality traits represents a potential answer to explain the fact that several elite and amateur athletes resort to doping substances. In 2017, a UK study (59) hypothesized for the first time the relationship of APED use in sport with Machiavellianism, narcissism, and psychopathy, the so-called “dark triad” personality traits, which leads to risky behaviors (60). Indeed, since Machiavellianism involves strategic orientation, it can be associated with doping use which indicates a sort of strategic thinking (e.g., doping is useful to achieve important goals). In addition, doping constitutes a “reckless” attitude, which is the key characteristic of psychopathy. Even if narcissism does not appear to be strictly related to APED use in sport, perfectionism does play a role in the decision to resort to performance-enhancing drugs (34). As a matter of fact, it was found that greater attitude in using APEDs by athletes was related to higher perfectionism scores in the Perfectionism in Sport Scale test.

In addition, parental and coach pressure toward perfectionism in young athletes generally entails a higher tendency to resort to performance-enhancing drugs, whereas perfectionistic efforts showed an inverse correlation (33).

For this reason, it is safe to assume that perfectionism can represent both a risk and protective factor against doping use, while self-control was demonstrated to be negatively associated with doping attitudes (61).

In this concern, an investigation carried out in Norway concluded that athletes consuming APEDs were neurotic, less open to experience, and less agreeable than non-consumers by non-athletes considering the personality traits of doped athletes based on their behavior (spontaneous trait inference approach) (62).

The last element to be taken into account involves individual differences in intrinsic and extrinsic motivation. Specifically, whereas intrinsic motivation is driven by autonomy and personal interest, the extrinsic one is characterized by external approvals. Consequently, meta-analyses have shown that with respect to extrinsic motivation, intrinsic motivation is associated with a lower likelihood to use APEDs in sports and increased effort, persistence, and satisfaction in the discipline (63, 64).

A different approach has been followed in the investigations of the reasons that drive recreational athletes toward the use of APEDs in non-competitive sport disciplines. In this case, the main motivation at the root of substance consumption is the improvement of body image and self-esteem stemming from success in outperforming others and prevailing. Indeed, a more recent study in five European countries showed that the main reasons for using prohibited APEDs among young non-competitive athletes and exercisers included the desire to achieve results faster, pushing oneself to the limits, and faster recovery after training (65).

## Personality Traits in Chemsexers Using APEDs

In reference to the array of drugs used to boost sexual performance, the term “chemsex” has been specifically coined to define the conscious or unconscious intake of psychoactive and non-psychoactive drugs to favor and/or stimulate sexual encounters mostly among men who have sex with other men (MSM) (11).

Drugs commonly associated with chemsex are mainly psychoactive drugs such as methamphetamine (also called crystal meth, the most used one), gamma hydroxybutyric acid and its precursor gamma butyrolactone, cocaine, ecstasy, cannabis, mephedrone, ketamine, and nitrates (poppers), together with erectile dysfunction medications (e.g., sildenafil, vardenafil, tadalafil). This evidence points out that chemsexers are akin to abusers of psychotropic drugs, likely presenting, in this way, the personality traits of drug addicts (66).

Chemsexers, frequently MSM, reportedly present the following characteristics: higher education level, higher income, smoking, sexual promiscuity, longer sexual activity duration, higher frequency of sexual intercourse, and lower sexual satisfaction levels (67).

With respect to the personality traits presented by chemsexers, it is difficult to distinguish if characteristic patterns of thoughts, feelings, and behaviors are derived from their specific personality or from polydrug use.

In an online survey performed on German chemsexers, they showed significantly higher mean scores for anxiety, depression, and somatization compared with non-chemsexers. Some men in the chemsex group experienced potentially adverse consequences, such as loss of control arising from negative impacts on social functioning, psychotic symptoms (13.2%), time and money spent for chemsex activities or amount of substances used at one occasion, and physically aggressive behavior toward others.

Similarly, an online questionnaire completed by Dutch MSM practicing chemsex evidenced that poor mental health levels, often due to anxiety and depression experienced by these individuals, were associated with drug addiction, loneliness, and HIV infection (68).

Depression and anxiety are two personality traits typical of MSM chemsexers. Indeed, behavioral characteristics, as described above for German and Dutch chemsexers, have been confirmed in Norwegian MSM as well and in other men participating in a cross-sectional clinic survey (69).

Conversely, Italian chemsexers declared to have consumed illicit drugs in order to intensify and prolong their sexual activity, keeping control on drug use. In that regard, the alleged “perceived control” could be considered among the criteria to discriminate between “problematic” vs. “recreational” chemsex behaviors and consequently associating quality of life impairment (70).

Generally speaking, all the above-reported observations highlight a link between chemsex in sexual intercourses and a psychological negative impact on MSM, whereby mental health, drug use, and HIV transmission risks overlap, partly driven by the psychosocial vulnerabilities experienced by chemsexers, who first started engaging in such practices as a coping strategy in extremely negative periods of stress, loneliness, and depressive mood. Undoubtedly, MSM experience higher rates of mental health problems compared with other men.

Furthermore, to complete all the above-reported concepts, qualitative data from a systematic review suggest that the personality traits promoting chemsex-related behaviors are as follows: coping with stressful events and painful emotions, risk trivializing sexualized drug use, giving into interpersonal pressure or fulfilling one's desire for community belonging, increasing intimacy, and lessening interpersonal and sexual inhibitions in terms of enhancing sexual performance and functioning (71).

To summarize, the use of sexual performance-enhancing drugs, such as the ones reported above among chemsexers, emerges from some fundamental traits such as an individual's desire to disengage from the cognitive burden generated by dissonant feelings; the knowledge that certain environments or pharmacologically active drugs will facilitate this disengagement; escaping from stress, anxiety, and depression; and attributing to substance use positive emotional and sexual experiences.

## Conclusion and Future Directions

As summarized for the above-reported subpopulation of APED users, the mechanisms underlying this behavior involve escape from stressful circumstances, anxiety, depression, body image disorders, dysfunctional diet and exercise patterns, and finally, personality traits attributable to the “dark triad” of personality—Machiavellianism, narcissism, and psychopathy—and perfectionism. It is worth bearing in mind that while Machiavellianism refers to interpersonal strategies aimed at furthering self-interest, deception, and manipulation, narcissism defines a pathological form of self-love, and lastly, psychopathy reflects superficial charm and remorselessness, selfishness, callousness, lack of interpersonal affection, and antisocial lifestyle and behaviors.

Problematic APED use has typically relied heavily on the classic addiction model of drug abuse and dependence. The difference with drug addicts lies in the fact that classic drug dependence entails a worsening of physical and occupational performance, whereas in APED consumers, physical, and professional capabilities and healthy lifestyle can be enhanced.

In any case, APED use remains a complex phenomenon that will require a specific set of diagnostic criteria to catch users who suffer from the consequences of such misuse behaviors and to develop clinical interventions and understanding of the etiology, course, and outcomes of APED use (1).

Nevertheless, it has been proposed that three criteria consisting of APED misuse, body image disturbance, and disorders affecting diet and exercise patterns can fall into a common standardized set of criteria relative to drug dependence (1). According to this proposal, initiatives and treatments need to coincide with those targeted to drug dependence. There have been several proposed alternatives to the longstanding drug-based model of APED addiction. The first one, developed by Kanayama and colleagues (25), incorporates body image dissatisfaction and compulsive exercise into the diagnostic criteria for this specific addiction. This approach allows for some degree of flexibility on the part of the clinician when assessing the larger set of phenomena typically associated with APED use, but maintains the same structure and criteria as the existing diagnostic criteria for other drug-based disorders. Overall, such a proposal keeps pathological APED use tied to the nosology of addiction. A more ambitious proposal, however, builds on the existing observational data and is based on the clustering of these three criteria that reflect the most impaired individuals who use APEDs. This proposal by Hildebrandt et al. (1) marks a unique blending of addiction, body image disturbance, and eating disorder nosology, thus highlighting the intersection of these types of psychopathologies in a specific subgroup of individuals. Epidemiological studies on APED users should be implemented, emphasizing the possible correlations with body image misperceptions and eating disorders.

In conclusion, APED use among the general population appears to be escalating into a major public health concern. It is therefore of paramount importance to lay out an objective assessment of their use by toxicological analyses (72, 73) and, consequently, to foster prevention and intervention measures for the purpose of promoting a healthy and tenable perception and use of one's body, not only for athletes but also among the general population, and to promote psychological aid procedures for people with substance use issues and body image disorders (74).

## Author Contributions

All authors listed have made a substantial, direct, and intellectual contribution to the work, and approved it for publication.

## Funding

This study has been funded by the Italian Department of Antidrug Policies.

## Conflict of Interest

The authors declare that the research was conducted in the absence of any commercial or financial relationships that could be construed as a potential conflict of interest. The reviewer SP declared a shared affiliation with one the authors AM at the time of the review and past co-authorship/collaboration with one or more authors AM, SZ, and EM.

## Publisher's Note

All claims expressed in this article are solely those of the authors and do not necessarily represent those of their affiliated organizations, or those of the publisher, the editors and the reviewers. Any product that may be evaluated in this article, or claim that may be made by its manufacturer, is not guaranteed or endorsed by the publisher.
